# CD147-spike protein is a novel route for SARS-CoV-2 infection to host cells

**DOI:** 10.1038/s41392-020-00426-x

**Published:** 2020-12-04

**Authors:** Ke Wang, Wei Chen, Zheng Zhang, Yongqiang Deng, Jian-Qi Lian, Peng Du, Ding Wei, Yang Zhang, Xiu-Xuan Sun, Li Gong, Xu Yang, Lei He, Lei Zhang, Zhiwei Yang, Jie-Jie Geng, Ruo Chen, Hai Zhang, Bin Wang, Yu-Meng Zhu, Gang Nan, Jian-Li Jiang, Ling Li, Jiao Wu, Peng Lin, Wan Huang, Liangzhi Xie, Zhao-Hui Zheng, Kui Zhang, Jin-Lin Miao, Hong-Yong Cui, Min Huang, Jun Zhang, Ling Fu, Xiang-Min Yang, Zhongpeng Zhao, Shihui Sun, Hongjing Gu, Zhe Wang, Chun-Fu Wang, Yacheng Lu, Ying-Ying Liu, Qing-Yi Wang, Huijie Bian, Ping Zhu, Zhi-Nan Chen

**Affiliations:** 1 National Translational Science Center for Molecular Medicine & Department of Cell Biology, Fourth Military Medical University, Xi’an, 710032 China; 2 Beijing Institute of Biotechnology, Beijing, 100071 China; 3 State Key Laboratory of Pathogen and Biosecurity, Beijing Institute of Microbiology and Epidemiology, Beijing, 100071 China; 4 Tangdu Hospital, Fourth Military Medical University, Xi’an, 710038 China; 5 MOE Key Laboratory for Nonequilibrium Synthesis and Modulation of Condensed Matter, Xi’an Jiaotong University, Xi’an, 710049 China; 6 Sino Biological Inc., Beijing, 100176 China; 7 Department of Clinical Immunology, Xijing Hospital, Fourth Military Medical University, Xi’an, 710032 China; 8 School of Basic Medicine, Fourth Military Medical University, Xi’an, 710032 China

**Keywords:** Industrial microbiology, Infection

## Abstract

In face of the everlasting battle toward COVID-19 and the rapid evolution of SARS-CoV-2, no specific and effective drugs for treating this disease have been reported until today. Angiotensin-converting enzyme 2 (ACE2), a receptor of SARS-CoV-2, mediates the virus infection by binding to spike protein. Although ACE2 is expressed in the lung, kidney, and intestine, its expressing levels are rather low, especially in the lung. Considering the great infectivity of COVID-19, we speculate that SARS-CoV-2 may depend on other routes to facilitate its infection. Here, we first discover an interaction between host cell receptor CD147 and SARS-CoV-2 spike protein. The loss of CD147 or blocking CD147 in Vero E6 and BEAS-2B cell lines by anti-CD147 antibody, Meplazumab, inhibits SARS-CoV-2 amplification. Expression of human CD147 allows virus entry into non-susceptible BHK-21 cells, which can be neutralized by CD147 extracellular fragment. Viral loads are detectable in the lungs of human CD147 (hCD147) mice infected with SARS-CoV-2, but not in those of virus-infected wild type mice. Interestingly, virions are observed in lymphocytes of lung tissue from a COVID-19 patient. Human T cells with a property of ACE2 natural deficiency can be infected with SARS-CoV-2 pseudovirus in a dose-dependent manner, which is specifically inhibited by Meplazumab. Furthermore, CD147 mediates virus entering host cells by endocytosis. Together, our study reveals a novel virus entry route, CD147-spike protein, which provides an important target for developing specific and effective drug against COVID-19.

## Introduction

The emergence of SARS-CoV-2 and its rapid spread across the world have triggered a global health emergency. As of 30 October 2020, there has been an outbreak of COVID-19 around the world, with 44,888,869 confirmed cases and 1,178,475 death cases. Patients with COVID-19 show abnormal findings on chest computed tomography, along with common symptoms that include cough, fever, and fatigue.^1^ Middle-aged and elderly patients with underlying comorbidities are susceptible to respiratory failure and may have a poorer prognosis.^2^ The pathological features of patients with COVID-19 are very similar to those of patients with SARS and MERS infection.^3^ Genetic mutation such as D614G in SARS-CoV-2 spike protein elevated COVID-19 infectivity.^4^ Until today no effective drugs are found for treating COVID-19. Therefore, intensive researches are urgently needed to elucidate the mechanisms of virus infection, in an attempt to provide a promising target for developing specific drug against COVID-19.

The primary determinant of coronavirus tropism is spike protein, which mediates the viral infection by binding to membrane receptors on the host cells.^5^ ACE2, a homologue of ACE, is one of the important receptors on the cell membrane of the host cells, which mediates SARS-CoV-2 infection for host cells by recognition of spike protein.^6,7^ ACE2 is reported to be expressed in the liver, lung, stomach, kidney, ileum, and colon, however, its expressing levels are rather low, especially in the lung.^8^ Considering the great infectivity of COVID-19, we speculate that SARS-CoV-2 may depend on other potential receptors to facilitate its infection. Recently, neuropilin-1 is identified as a co-factor involved in ACE2-mediated SARS-CoV-2 infection.^9,10^ However, other routes for SARS-CoV-2 infection remain unclear.

CD147, known as *basigin* or EMMPRIN, is a transmembrane glycoprotein of the immunoglobulin superfamily,^11^ which participates in tumor development, *Plasmodium* invasion, and bacterial and virus infection.^12–16^ Our previous studies show that CD147 plays a functional role in facilitating SARS-CoV infection, and CD147-antagonistic peptide-9 has an inhibitory effect on SARS-CoV.^17^ These works affirm the importance of CD147 in virus infection for host cells. Based on the previous study, we conduct the present study to investigate the possible function of CD147 in SARS-CoV-2 infection.

In our study, we report a direct interaction of CD147 and SARS-CoV-2 spike protein, which mediates virus infection for host cells. The loss of CD147 or blocking CD147 by Meplazumab inhibits SARS-CoV-2 replication; by contrast CD147 overexpression promotes virus infection. Viral loads are detectable in the lungs of hCD147 mice infected with SARS-CoV-2. Moreover, SARS-CoV-2 virions enter the host cells through the CD147-spike protein route by endocytosis. These results reveal a new receptor for virus entry, which is of great importance for developing specific and effective drug in the treatment of COVID-19.

## Results

### CD147 interacts with SARS-CoV-2 spike protein

**Fig. 1:**
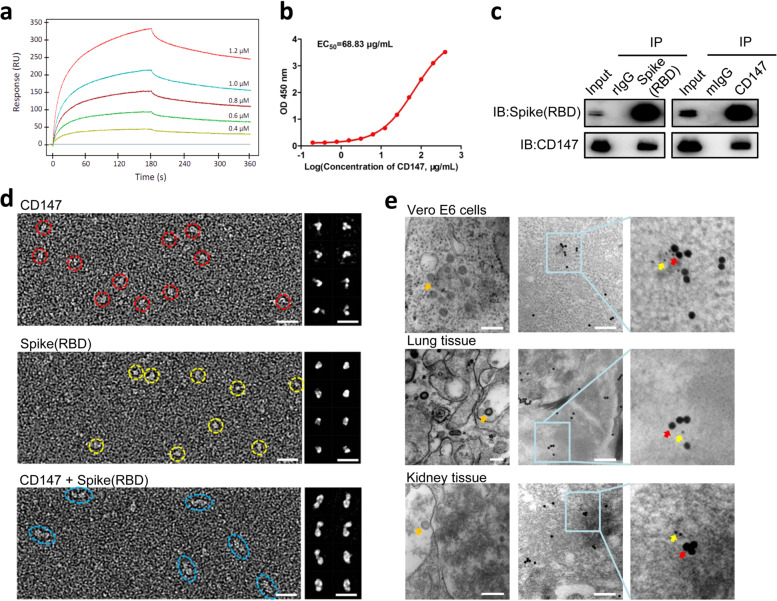
Identification of the interaction and co-localization between CD147 and spike protein. **a**–**c** The interaction of CD147 and spike was detected by SPR assay (**a**), ELISA (**b**), and Co-IP assay (**c**). The mouse IgG (mIgG) and rabbit IgG (rIgG) were served as negative controls. **d** OpNS-EM images of CD147, spike(RBD) and CD147-spike(RBD) complexes. Left panels showed the survey view of the micrograph. Right panels showed 8 class averages were selected from a total of more than 300 class averages which were respectively calculated from a total of 6,681 CD147 particles; 5,073 particles of spike(RBD); 12,426 particles of CD147-spike(RBD) complexes. Scale bars: 10 nm. **e** The co-localization of CD147 and spike protein was observed by immuno-electron microscope. Virions (orange arrows) were observed in virus-infected Vero E6 cells and lung and kidney tissues from COVID-19 patient. The co-localization of CD147 (20 nm-gold colloid, red arrows) and spike protein (10 nm-gold colloid, yellow arrows) in SARS-CoV-2 infected Vero E6 cells and lung and kidney tissues from a patient with COVID-19. Scale bars: 200 nm

To investigate whether CD147 involves in SARS-CoV-2 infection, surface plasmon resonance (SPR) and enzyme-linked immunosorbent assay (ELISA) were performed and showed an interaction between CD147 and spike(RBD), with an affinity constant of 1.85 × 10^–7^ M (Fig. 1a) and half-maximal effective concentration (EC_50_) of 68.83 μg/mL (Fig. 1b). Co-IP analysis confirmed the interaction between CD147 and spike(RBD) (Fig. 1c). Optimized negative-staining electron microscopy (OpNS-EM) showed that the CD147 protein appeared as a folded stick with a junction in the middle, while the spike(RBD) protein appeared near-spherical structures with a diameter of 3 nm. For CD147-spike(RBD) mixture, the class-averages exhibited two domains, which had consistent shapes with a single CD147 and a single spike(RBD), indicating binaries of CD147 and spike(RBD) (Fig. 1d). In addition, electron microscope observed virions in SARS-CoV-2-infected Vero E6 cells, as well as lung and kidney tissues from a patient with COVID-19. After traced by 20 nm (CD147) and 10 nm (spike) gold colloid-labeled antibodies, the co-localization of CD147 and spike was found in Vero E6 cells, and lung and kidney tissues (Fig. 1e). These results verify the interaction between CD147 and spike protein, which may mediate virus infection for host cells.

### SARS-CoV-2 employs the CD147 receptor for host cell entry

**Fig. 2:**
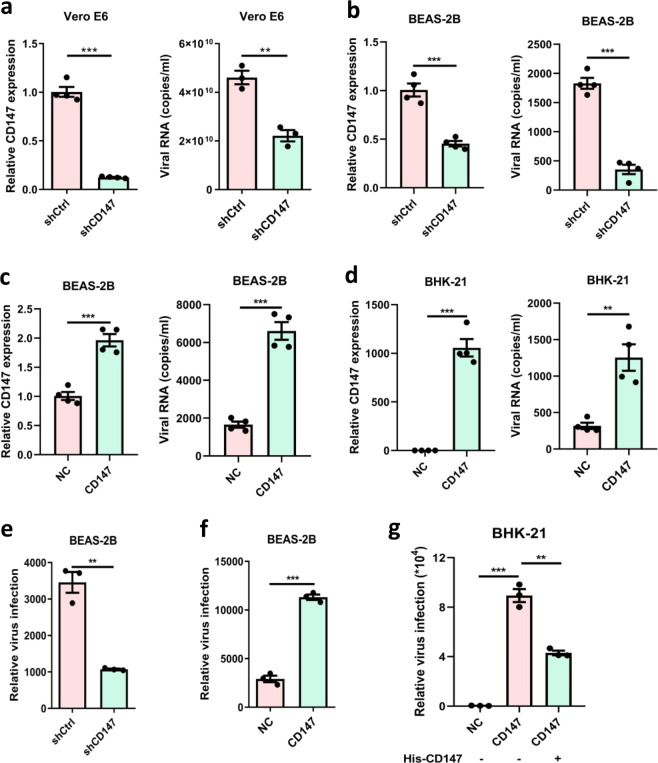
SARS-CoV-2 employs the CD147 receptor for host cell entry. **a**, **b** Left, Vero E6 and BEAS-2B cells were transfected with shRNA for CD147 gene silence; the gene expression level was detected by real-time PCR. Right, SARS-CoV-2 infection test was performed in Vero E6-shCD147 and BEAS-2B-shCD147 cells. At 48 h after infection, the virus copy number was detected with quantitative PCR (**p < 0.01, ***p < 0.001, two-tailed *t*-test, mean ± SEM). **c**, **d** Left, CD147 expression level was detected by real-time PCR in BEAS-2B-CD147 and BHK-21-CD147 cells. Right, SARS-CoV-2 infection test was performed in BEAS-2B-CD147 and BHK-21-CD147 cells. At 48 h after infection, the virus copy number was detected with quantitative PCR (**p < 0.01, ***p < 0.001, two-tailed *t*-test, mean ± SEM). **e**, **f** The infection efficiency of SARS-CoV-2 pseudovirus was detected by luciferase reporter assay in BEAS-2B-shCD147 and BEAS-2B-CD147 cells (**p < 0.01, ***p < 0.001, two-tailed *t*-test, mean ± SEM). **g** SARS-CoV-2 pseudovirus infection of BHK-21-CD147 cells was neutralized by recombinant human CD147 extracellular fragment (**p < 0.01, ***p < 0.001, two-tailed *t*-test, mean ± SEM)

**Fig. 3:**
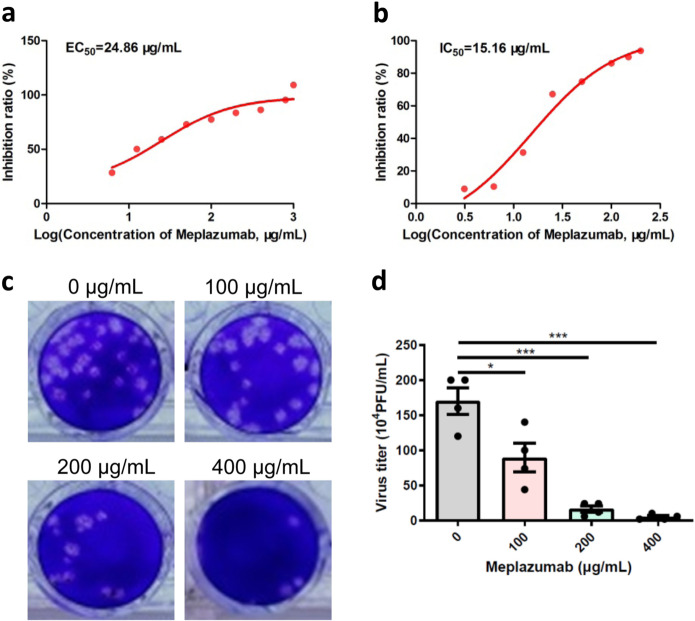
Meplazumab significantly inhibited virus replication. **a**, **b** Virus inhibition assays were performed to calculate EC_50_ and IC_50_ by crystal violet staining and quantitative PCR, respectively. **c**, **d** The inhibitory effect of Meplazumab on virus infection was detected by plaque assay and virus titer was quantified (*p < 0.05, ***p < 0.001, two-tailed *t*-test, mean ± SEM)

To explore the role of CD147 in SARS-CoV-2 infection, CD147 stable knockdown cells, Vero E6-shCD147, and BEAS-2B-shCD147, were infected with virus for 48 h. Quantitative PCR analysis showed that virus copy number was markedly decreased in CD147 knockdown group (Fig. 2a, b). In contrast, CD147 overexpression in BEAS-2B cells promoted virus infection (Fig. 2c). More importantly, the expression of human CD147 allows SARS-CoV-2 to enter into non-susceptible BHK-21 cells (Fig. 2d). The consistent findings were obtained in BEAS-2B-shCD147, BEAS-2B-CD147, and BHK-21-CD147 cells using SARS-CoV-2 pseudovirus (Fig. 2e–g). Moreover, human CD147 extracellular fragment inhibited pseudovirus entry into BHK-21-CD147 cells, suggesting the fragment could compete with membrane receptor CD147 when binding to spike protein (Fig. 2g). In the meanwhile, the virus-induced cytopathic effect was inhibited by an anti-CD147 antibody, Meplazumab, in a dose-dependent manner with EC_50_ of 24.86 μg/mL (Fig. 3a). The virus copy number was also suppressed by Meplazumab with half-maximal inhibitory concentration (IC_50_) of 15.16 μg/mL (Fig. 3b). Plaque assay demonstrated that Meplazumab significantly inhibited virus replication (Fig. 3c, d). These results prove CD147 functions as a cellular receptor for SARS-CoV-2 entering host cells.

### CD147 mediates SARS-CoV-2 infection in hCD147 mice

**Fig. 4:**
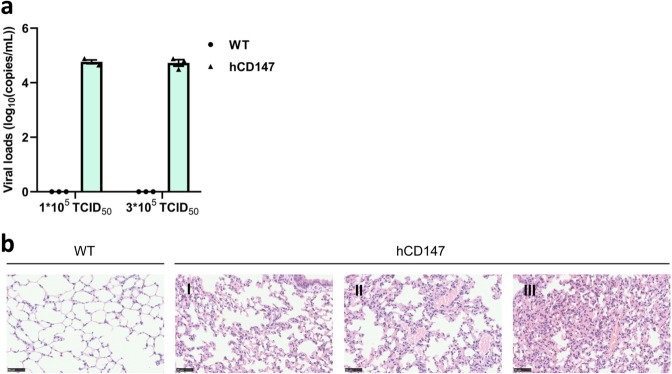
SARS-CoV-2 invades lung tissues of hCD147 mice and causes pathologic changes. **a** The WT mice and hCD147 mice were infected with SARS-CoV-2. At 48 h after infection, the viral load of lung tissues was detected with quantitative PCR. **b** The histopathological changes of lung tissues were detected in WT mice and hCD147 mice by HE staining. Scale bars: 50 μm

The hCD147 mice were constructed to investigate the infection efficiency of SARS-CoV-2. As shown in Fig. 4a, viral loads were detectable in the lungs of hCD147 mice infected with SARS-CoV-2, but not in those of virus-infected wild-type mice at 48-h post infection. Hematoxylin & eosin (HE) staining showed the hCD147 mice developed pneumonia with alveolar septal thickening (I), serofluid exudation, hyaloid membrane formation (II), inflammatory cell infiltration, and pulmonary consolidation (III), but there were no obviously histopathological changes in wild-type mice (Fig. 4b). These findings indicate that the virus-susceptible mouse model is successfully constructed and reinforce the significant role of CD147 as a receptor in SARS-CoV-2 infection.

### CD147 is an alternative receptor for SARS-CoV-2 infection in ACE2-deficient cell types

**Fig. 5:**
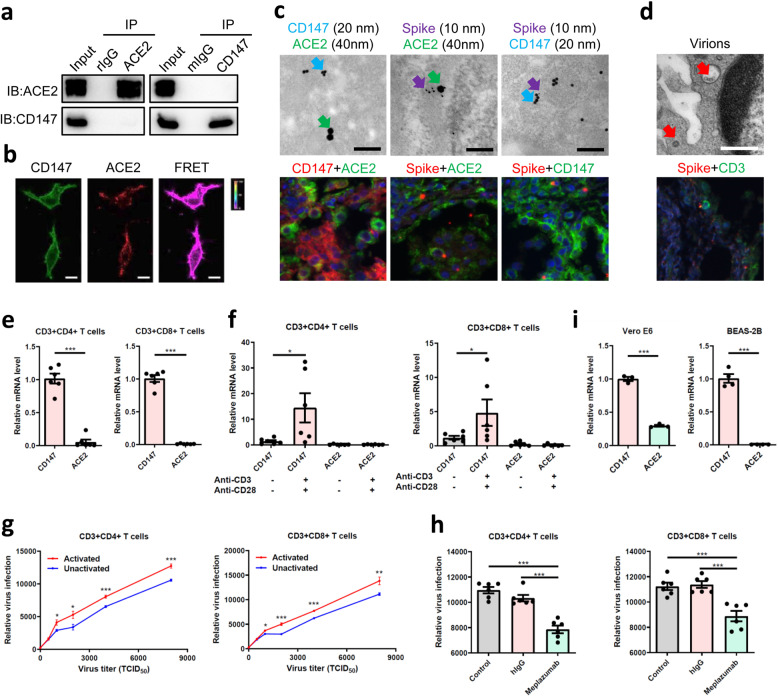
CD147 is an alternative receptor for SARS-CoV-2 infection in ACE2-deficient cell types. **a** No interaction of CD147 and ACE2 was detected by Co-IP assay. The mIgG and rIgG were served as negative controls. **b** No co-localization was found between CD147 and ACE2 by FRET. The color bar denotes FRET ratio. Scale bars: 10 μm. **c** No co-localization of CD147-ACE2 and the co-localizations of spike-ACE2 and spike-CD147 were observed by immuno-electron microscope (scale bars: 200 nm) and multicolor immunofluorescence (magnification: ×200) in lung tissues from COVID-19 patient. Spike protein, 10 nm-gold colloid, purple arrows; CD147, 20 nm-gold colloid, blue arrows; and ACE2, 40 nm-gold colloid, green arrows. **d** Virions (red arrows) were observed in lymphocytes of lung tissues from COVID-19 patient. Scale bars: 500 nm. The localization of spike protein and CD3 was analyzed by multicolor immunofluorescence staining. Magnification: ×200. **e** The gene expressions of CD147 and ACE2 in CD4+ and CD8+ T cells were detected by real-time PCR (***p < 0.001, two-tailed *t*-test, mean ± SEM). **f** The expressions of CD147 and ACE2 in unactivated or activated T cells were detected by real-time PCR (*p < 0.05, Mann–Whitney test, mean ± SEM). **g** The infection efficiency of SARS-CoV-2 pseudovirus was detected by luciferase reporter assay in unactivated and activated T cells (*p < 0.05, **p < 0.01, ***p < 0.001, two-tailed *t*-test, mean ± SEM). **h** SARS-CoV-2 pseudovirus infection of CD4+ and CD8+ T cells was inhibited by Meplazumab (***p < 0.001, two-tailed *t*-test, mean ± SEM). **i** The gene expressions of CD147 and ACE2 in Vero E6 and BEAS-2B cells were detected by real-time PCR (***p < 0.001, two-tailed *t*-test, mean ± SEM)

As both CD147 and ACE2 contribute to virus infection for host cells by binding to spike protein, we wonder how CD147 works alongside ACE2. We found no interaction between CD147 and ACE2 by Co-IP assay (Fig. 5a), which was verified by fluorescence resonance energy transfer (FRET) assay (Fig. 5b). Furthermore, the co-localizations between CD147 and spike protein, and ACE2 and spike protein were detected in lung tissues from patient with COVID-19, but no co-localization between CD147 and ACE2 was observed (Fig. 5c). Previous study provides the evidences that lymphocytes are infected and injured by SARS-CoV, leading to decreased circulating lymphocytes.^18^ Interestingly, in our study, SARS-CoV-2 virions were observed in lymphocytes in lung tissue from COVID-19 patient (Fig. 5d). T cells derived from human peripheral blood mononuclear cells (PBMC) were collected to analyze the expressions of CD147 and ACE2 by real-time PCR assay, which demonstrated that CD147 expression was detected in CD4+ and CD8+ T cells, but no expression of ACE2 was detected (Fig. 5e). It has been reported that the destruction of lung cells by SARS-CoV-2 infection triggers inflammatory storm, including T cell immune responses.^19^ CD147 is also reported to be significantly upregulated in activated T lymphocytes.^20^ Here, we confirmed that the expression of CD147 was significantly increased in both activated CD4+ and CD8+ T cells, however, the expression of ACE2 was not increased (Fig. 5f). Virus infection assays showed that T cells were infected with SARS-CoV-2 pseudovirus in a dose-dependent manner, and significantly enhanced infection of pseudovirus was observed in activated T cells, compared to unactivated T cells (Fig. 5g). Moreover, Meplazumab significantly inhibited the pseudovirus from infecting T cells (Fig. 5h). These results indicate that CD147 is an alternative receptor for SARS-CoV-2 infection, especially in ACE2-deficient cell types. It has been reported that CD8+ T cell decline by SARS-CoV-2 infection is related to a poor prognosis and systemic inflammation in patients with COVID-19.^21^ Our results provide the evidence that CD147 mediates infection of SARS-CoV-2 in CD4+ and CD8+ T cells, exerting significant impacts on the prognosis of patients. In the meanwhile, the expressions of CD147 and ACE2 were also analyzed in human bronchial epithelial cells, BEAS-2B, and Vero E6 cells. As shown in Fig. 5i, the expression level of CD147 was 2.33-fold higher than that of ACE2 in Vero E6 cells and no expression of ACE2 was detected in BEAS-2B cells. These findings demonstrate that CD147 provides more entries for SARS-CoV-2 infection and plays a potential role in mediating virus infection, especially in ACE2-deficient cell types.

### SARS-CoV-2 enters the host cells through CD147-mediated endocytosis

**Fig. 6:**
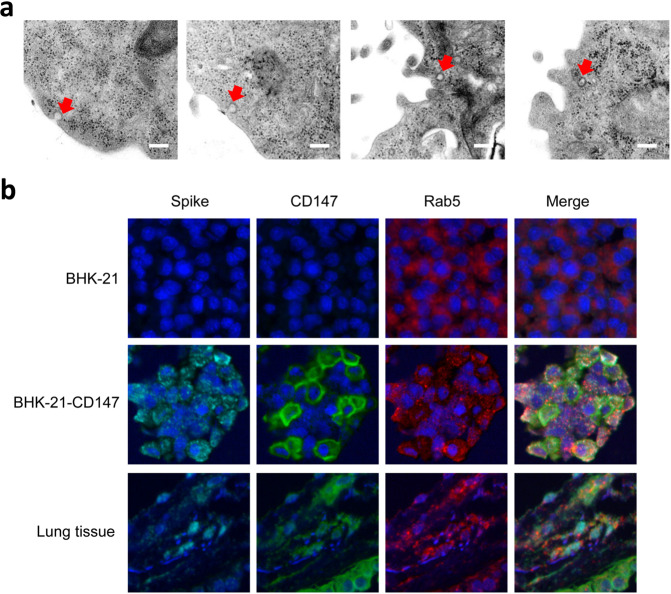
SARS-CoV-2 enters the host cells through CD147-mediated endocytosis. **a** The sequential endocytosis of SARS-CoV-2 was observed in Vero E6 cells by electron microscope. Scale bars: 200 nm. **b** The co-localization of spike protein, CD147, and Rab5 were analyzed in BHK-21-CD147 cells and lung tissues from COVID-19 patient by multicolor immunofluorescence staining. Magnification: ×200.

It is reported that membrane fusion and endocytosis are two main entry modes for virus infection.^22,23^ Here, we found a sequential endocytosis of SARS-CoV-2 in Vero E6 cells by electron microscope (Fig. 6a). CD147 is reported to enter the cells through clathrin-independent endocytosis.^24,25^ Rab5 is a crucial regulator of endocytosis and locates at early endosome.^26^ In our study, the co-localization of CD147, spike, and Rab5 was detected in BHK-21-CD147 cells and lung tissues from patient with COVID-19 (Fig. 6b), indicating that the receptor CD147 and virions were endocytosed and located at the early endosome. Taken together, SARS-CoV-2 enters the host cells through CD147-mediated endocytosis.

## Discussion

The COVID-19 pandemic has become a primary public health issue across the globe, causing severe respiratory tract infections in humans. Due to the interventions and control measures and the change in personal behaviors, the confirmed and suspected cases have been decreased in certain areas.^27^ However, in most countries and regions, SARS-CoV-2 is still widely spread with substantial morbidity and mortality, which call for safe and effective drugs against COVID-19. So far no specific and effective drugs for treating this disease have been reported.

ACE2 has been widely recognized as a receptor in coronavirus infection, and SARS-CoV replication is specifically inhibited by an anti-ACE2 antibody.^28^ However, the presence of ACE2 in the heart, kidney, and testis^29^ play a profound role in controlling blood pressure and preventing heart failure and kidney injury.^30–32^ For lung diseases, the loss of ACE2 activates the renin-angiotensin system, enhancing vascular permeability and lung edema and contributing to the pathogenesis of severe lung injury.^33^ The entry of SARS-CoV-2 into the cells markedly down-regulates ACE2 receptors, which favors the progression of inflammatory and thrombotic processes.^34^ Therefore, treatment by blocking ACE2 receptor probably has a negative effect and poor druggability.

In our study, we discover CD147 as a new receptor for SARS-CoV-2 infection. It has been reported that BHK-21 cells cannot be infected with SARS-CoV-2,^7^ which is also confirmed in our study. However, the introduction of CD147 alters the virus tropism toward BHK-21 cells. CD147 as a new receptor is also approved by loss and gain function approaches and neutralization activity of CD147 extracellular domain. Another meaningful finding in our work is that ACE2-deficient T cells can be infected with SARS-CoV-2 pseudovirus, in which CD147 overexpression facilitates the virus infection. Previously, the SARS-CoV virions and genomic sequence were analyzed in a great number of peripheral lymphocytes, causing the destruction of lymphocytes.^18^ Our finding offers a possible explanation for lymphopenia in COVID-19 cases.^35^ The expression of CD147 is reported to be elevated in inflammatory alveolar epithelial cells,^36^ and ACE2 is down-regulated by SARS-CoV infection.^37^ In the meanwhile, the expression level of CD147 is higher than that of ACE2 in Vero E6 cells and no expression of ACE2 is detected in BEAS-2B cells. Interestingly, immunofluorescent assay shows no co-localization of CD147 and ACE2 in lung tissues from COVID-19 patient, as well as no interaction in detected cells. The expressions of CD147 and ACE2 are exclusively independent in single lung cell. These findings suggest that CD147 and ACE2 may be two complementary receptors in mediating virus infection.

In conclusion, our study discovers a novel virus entry route, CD147-spike protein, which sheds light on a new mechanism of SARS-CoV-2 infection and provides a specific therapeutic strategy in COVID-19 treatment.

## Materials and methods

### Ethics statement

The study was approved by the ethics committees of Xijing Hospital and Tangdu Hospital, Fourth Military Medical University (KY20202005-1, K202002-01, E202003-01). SARS-CoV-2 strain (BetaCov/human/CHN/Beijing_IME-BJ05/2020) for cell experiments was isolated and preserved in the State Key Laboratory of Pathogen and Biosecurity at Beijing Institute of Microbiology and Epidemiology. SARS-CoV-2 strain (BetaCoV/Wuhan/IVDC-HB-01/2020|EPI_ISL_402119) was used for mice experiments in the Chinese Academy of Medical Sciences.

### Cell culture

Vero E6, BEAS-2B, BHK-21, and HEK293T cell lines were from the Cell Bank of the Chinese Academy of Sciences (Shanghai, China). All the cell lines were authenticated using Short Tandem Repeat DNA profiling by Beijing Microread Genetics Co., Ltd (Beijing, China) and were cultured at 37 °C under 5% CO_2_ in DMEM or RPMI 1640 medium supplemented with 10% fetal bovine serum (FBS), 1% penicillin/streptomycin, and 2% L-glutamine.

### Meplazumab

Meplazumab is a humanized IgG2 antibody targeting CD147, which is provided by Jiangsu Pacific Meinuoke Biopharmceutical Co. Ltd, China.

### In vitro antiviral tests

Vero E6 cells (1 × 10^4^) were cultured in a 96-well plate at 37 °C overnight, the supernatant was discarded and 100 μl of medium (containing 6.25, 12.5, 25, 50, 100, 200, 400, 800, 1000 μg/mL Meplazumab) was added into the plates to incubate for 1 h. Then the cells were infected with SARS-CoV-2 (100 Median Tissue Culture Infectious Dose, TCID_50_). After 1 h infection, the supernatants were removed and 200 μl of medium (containing 6.25, 12.5, 25, 50, 100, 200, 400, 800, 1000 μg/mL Meplazumab) was added, the cells were cultured to observe the cytopathic changes for 3 days by crystal violet staining to calculate EC_50_. The same method was employed with different antibody concentration (containing 3.125, 6.25, 12.5, 25, 50, 100, 150, 200 μg/mL Meplazumab). The supernatants were harvested to detect the gene copy number of the virus with quantitative PCR to calculate IC_50_ (QIAampViral RNA Mini Kit, QIAGEN, Germany; One-Step RT-PCR kit, RR064A, Takara, Japan). A virus control group and a blank control group were set at the same time. The primers and probe sequences were listed as follows:

SARS-CoV-2-Forward: 5′-TCCTGGTGATTCTTCTTCAGGT-3′

SARS-CoV-2-Reverse: 5′-TCTGAGAGAGGGTCAAGTGC-3′

SARS-CoV-2-Probe: 5′-FAM-AGCTGCAGCACCAGCTGTCCA-BHQ1-3′

### Plaque assay

Vero E6 cells (1 × 10^5^) were cultured in a 24-well plate at 37 °C overnight, the supernatant was discarded and 300 μl of medium (containing 100, 200, 400 μg/mL Meplazumab) was added into the plates to incubate for 1 h. Then the cells were infected with SARS-CoV-2 (25 TCID_50_). After 1 h infection, the supernatants were removed and 500 μl of medium (containing 100, 200, 400 μg/mL Meplazumab) was added. The cells were cultured for 3 days and then the virus titer was detected by plaque assay.

### Generation of stable knockdown and overexpressed cell lines

The lentiviruses were purchased from GENECHEM Co. Ltd. The Vero E6, BEAS-2B, and BHK-21 cells were transfected by Lipofectamine 2000 reagent (Invitrogen, USA) with supernatant containing lentivirus carrying the shCD147 or CD147 construct to generate CD147 knockdown or overexpressed cell lines, named Vero E6-shCD147, BEAS-2B-shCD147, BEAS-2B-CD147, and BHK-21-CD147. After 48 h, the infected cells were selected with 3 μg/mL puromycin and a monoclonal cell was selected for further study.

### RNA extraction and real-time PCR analysis

The cells were collected to detect the expression of CD147 and ACE2 genes. The tests were performed according to the previous methods.^38^ The primer sequences were listed as follows:

Human CD147-Forward: 5′-GACGACCAGTGGGGAGAGTA-3′

Human CD147-Reverse: 5′-GGCCTTGTCCTCAGAGTCAG-3′

Monkey CD147-Forward: 5′-GGCTCGAAGACACTCCTCAC-3′

Monkey CD147-Reverse: 5′-GAGTACTCTCCCCCGAGGTC-3′

Human/monkey ACE2-Forward: 5′-GTGCACAAAGGTGACAATGG-3′

Human/monkey ACE2-Reverse: 5′-GGCTGCAGAAAGTGACATGA-3′

Human/monkey GAPDH-Forward: 5′-GCACCGTCAAGGCTGAGAAC-3′

Human/monkey GAPDH-Reverse: 5′-TGGTGAAGACGCCAGTGGA-3′

### In vitro virus infection test

Virus infection test was performed using CD147 knockdown or overexpressed cell lines. The cell medium was discarded and the virus (20 TCID_50_) was added into each reaction. After the cells infected for 1 h at 37 °C, the virus supernatant was discarded and the cells were washed twice with PBS. Finally, the cells were cultured with 2% FBS maintenance medium. At 48 h after infection, the QIAampViral RNA Mini Kit (QIAGEN, Germany) was used to extract the viral nucleic acid from the cell supernatant and the gene copy number of the virus was detected with quantitative PCR (One-Step RT-PCR kit, RR064A, Takara, Japan).

### In vitro SARS-CoV-2 pseudovirus infection test

SARS-CoV-2 pseudovirus with luciferase^39^ was obtained from Institute for Biological Product Control, National Institutes for Food and Drug Control (Beijing, China). CD4+ and CD8+ T cells were sorted from PBMCs derived from six healthy volunteers using APC/Cyanine7 anti-human CD3 antibody (300426, Biolegend, USA), PE/Cyanine7 anti-human CD4 antibody (357410, Biolegend, USA), and PerCP anti-human CD8a antibody (300922, Biolegend, USA). 1 × 10^6^ CD4+ or CD8+ T cells were activated by purified NA/LE mouse anti-human CD3 antibody (555329, BD, USA, 1 μg/mL) and purified NA/LE mouse anti-human CD28 antibody (555725, BD, USA, 2 μg/mL) for 48 h. Then, the different doses of SARS-CoV-2 pseudovirus (0, 500, 1000, 2000, 4000, and 8000 TCID_50_) were added into unactivated or activated CD4+ and CD8+ T cells and incubated for 72 h. In addition, the 8000 TCID_50_ group was also incubated with Meplazumab or human IgG to evaluate the inhibitory effect for SARS-CoV-2 pseudovirus infection. The system was detected using Dual-Luciferase® Reporter Assay System (E1960, Promega, USA) according to the manufacturer’s protocol. In the same way, the SARS-CoV-2 pseudovirus (5000 TCID_50_) was used to infect BEAS-2B-shCD147, BEAS-2B-CD147, and BHK-21-CD147 cells for 72 h, and 100 μg/mL His-CD147 was performed to incubate with pseudovirus for 1 h in BHK-21-CD147 group. The infection efficiency was also evaluated by Dual-Luciferase® Reporter Assay System.

### SPR assay

The receptor-binding domain (RBD) of spike protein specifically binding to ACE2 was obtained from GenScript. SPR analysis was performed by BIAcore 3000 system (BIAcore, USA). His-CD147 (produced by our laboratory) was fixed to the surface of CM5 sensor chips (GE Healthcare Bio-Sciences AB) by amino coupling kit (GE Healthcare, BR-1000-50). The interaction between CD147 and spike(RBD) was detected using Kinetic Analysis/Concentration Series/Direct Binding mode, and the flow rate was set to 15 μl/min, both binding time and dissociation time were 3 min. The results were analyzed by BIAevaluation software to determine the affinity constant.

### Co-IP assay

Co-IP assay was performed using Pierce^®^ Co-Immunoprecipitation Kit (26149, Thermo Fisher Scientific, USA) according to the manufacturer’s protocol. Mouse anti-human CD147 antibody (Jiangsu Pacific Meinuoke Biopharmceutical Co. Ltd, China, 50 μg), anti-SARS-CoV-2 spike antibody (40150-R007, Sino Biological, China, 50 μg) and anti-ACE2 antibody (80031-R003, Sino Biological, China, 50 μg) were used for antibody immobilization for Co-IP. The eluted proteins were detected by western blot. After boiling for 5–10 min, the eluted proteins were loaded to 12% SDS-PAGE gel and then transferred to PVDF membranes (Millipore, Boston, USA). After blocking with 5% non-fat milk for 1 h, the membrane was incubated with the corresponding primary antibodies at 4 °C overnight. The images were developed after incubation with the secondary antibodies (goat anti-mouse IgG(H + L) antibody, 31430, Thermo Fisher Scientific, MA, USA, dilution ratio 1:5000; goat anti-rabbit IgG(H + L) antibody; 31460, Thermo Fisher Scientific, MA, USA, dilution ratio 1:5000) at room temperature for 1 h.

### ELISA

ELISA assay was performed to identify the interaction of CD147 and spike. The His-spike(RBD) protein was coated on microplate, and then incubated with different concentrations of His-CD147 protein (twofold dilution, 400–0.195 μg/mL) at 37 °C for 1 h. After washing with PBST, the samples were incubated with mouse anti-human CD147 antibody (2 μg/mL) for 1 h, and then incubated with horseradish peroxidase HRP-labeled goat anti-mouse antibody (dilution ratio 1:6000) for 1 h. After coloration, the OD value at 450 nm was measured with a full-wavelength microplate reader (Epoch, BioTek Instruments, Inc., USA).

### OpNS-EM specimen preparation

Specimens were prepared for electron microscopy by the OpNS-EM protocol.^40^ In brief, CD147 (2.26 mg/mL) and spike(RBD) (1.04 mg/mL) were diluted to 0.02 and 0.01 mg/mL with PBS. By incubating CD147 and spike(RBD) with 1:1 molar ratio, i.e., 1.5 mg/mL of spike(RBD) and 1.5 mg/mL of CD147, at 4 °C for 4 h, the CD147-spike(RBD) complexes were formed and then diluted to a concentration of 0.015 mg/mL with PBS buffer. Then an aliquot (~3 μl) was placed on an ultra-thin carbon film-coated 200-mesh copper grid (Cu-200UL, Electron Microscopy Sciences, USA) which had been glow discharged. After ~1 min, excess CD147-spike(RBD) complexes solution was blotted with a piece of filter paper. Then the grid was quickly washed by touching the grid surface with a drop (~35 μl) of distilled water on parafilm and blotting dry with another piece of filter paper. Such touching and blotting step was repeated three times. For each time, a clean drop of clean distilled water was used. Then three drops of freshly prepared ~1% (w/v) uranyl formate (UF) negative stain (Electron Microscopy Sciences, USA) solution on parafilm were applied successively, and by blotting in the same way, excess stain solution was removed. The carbon-coated copper grid was then allowed to stay on the last drop of stain solution with the sample side down for ~1 min in a light-proof container. Finally, the excess stain solution was removed and the grid was air dried at room temperature.

### Electron microscopy data collection and image processing

OpNS-EM micrographs were acquired at room temperature on a FEI CETA 16 M CMOS camera by a FEI F200C TEM operating at ×120,000 magnification, 200 kV high-tension, and low-electron-dose conditions under a defocus in a range of 0.15–0.65 μm. Under such conditions, each pixel of the raw micrographs corresponded to 1.00 Å in the specimens. The collected TEM Images were then processed with EMAN, SPIDER, and FREALIGN software packages. The parameters of the contrast transfer function (CTF) for each raw micrograph were fitted and determined with ctffind3 in FREALIGN^41^ and then corrected with SPIDER software.^42^ For particle selecting, the e2boxer.py program in EMAN2^43^ were used, and then the automatically selected particles were examined and extracted with the EMAN package (boxer program).^44^ A 2D reference-free class-averaging program was then applied.^45^ After quality evaluation, particle images from each sample (6681 CD147; 5073 spike(RBD); 12,426 CD147-spike(RBD) complexes) were selected from the corrected micrographs and extracted as 128 × 128-pixel images by using boxer software.^44^ Then the contrast of the particle images was normalized to the same range after the X-ray sparkles were filtered out. For further and finer classification, the circular mask with a Gaussian type boundary was applied to all particle images. Then four iterations of classifications were performed by using refine2d.py software (EMAN package),^44^ and more than 300 classes/groups were finally obtained based on the values of cross-correlation coefficients. Finally, the images in each group were aligned to each other and averaged together to increase the signal-noise ratio.^44^

### Immuno-electron microscope

The lung and kidney tissues were collected from a patient with COVID-19 in Tangdu Hospital of Fourth Military Medical University. Vero E6 cells infected with SARS-CoV-2, and lung and kidney tissues were harvested and fixed at 4 °C. The sections were prepared through dehydration and embedding. After washing with ultrapure water, the sections were treated with 1% H_2_O_2_ for 10 min. Having been blocked with goat serum for 30 min, the sections were incubated with corresponding primary antibodies (rabbit anti-human CD147 antibody, produced by our laboratory; mouse anti-2019-nCoV Spike antibody, Sino Biological, China; mouse anti-ACE2 antibody, Proteintech, USA, rabbit anti-SARS-CoV-2 Spike antibody, 40150-R007, Sino Biological, China) for 16 h at 4 °C overnight. Subsequently, the sections were washed with PBS for 5 min, and treated with PBS (containing 1% bovine serum albumin, BSA, pH8.2) for 7 min. Then the gold colloid-labeling method (20 nm InnovaCoat® GOLD (10 OD) Goat Anti Rabbit IgG Conjugate, 219-1000, Expedeon; InnovaCoat® GOLD-40 nm Goat Anti Mouse nanoparticles, 216–1000, Expedeon; InnovaCoat® GOLD-10nm Goat Anti Rabbit nanoparticles, 218–1000, Expedeon; Anti-Mouse IgG (whole molecule)-Gold antibody produced in goat, affinity isolated antibody, aqueous glycerol suspension, 10 nm colloidal gold, G7777, Sigma-Aldrich) was used to determine the localization of targeted proteins for 1 h at room temperature, and the sections were consecutively stained with 5% uranium acetate and lead acetate. The protein location was observed by immune-electron microscope (JEM-1230, JEOLLTD, Japan).

### Fluorescence resonance energy transfer assay (FRET)

The two plasmids eGFP-N1-CD147 and DsRed-N1-ACE2 were co-transfected to HEK293T cells for 48 h. The FRET assay was performed by referring to the methods described previously.^46^ The FRET efficiency calculation and image processing were done by analysis software (Nikon, Japan).

### Mouse experiment

The animal experiments were performed in strict accordance with the People’s Republic of China Legislation Regarding the Use and Care of Laboratory Animals. All protocols used in this study were approved by the Institutional Animal Care and Use Committee (IACUC) of Fourth Military Medical University (20200206). Human CD147 (hCD147) mice were provided by Shanghai Model Organisms Center, Inc. (China). Extracellular region of CD147 in wild-type (WT) C57BL/6J mice was replaced with human CD147 by targeting embryonic stem cells. After receiving anesthesia by 2.5% avertin with 0.02 mL/g body weight, each mouse was inoculated intranasally with SARS-CoV-2 at a dose of 1 × 10^5^ TCID_50_ (WT, *n* = 3; hCD147, *n* = 3) and 3 × 10^5^ TCID_50_ (WT, *n* = 3; hCD147, *n* = 3). After 2 days, lung tissues were collected for virus loads detection and HE staining. The primer sequences for the envelope gene of SARS-CoV-2 were listed as follows:

Envelope gene-Forward: 5′-TCGTTTCGGAAGAGACAGGT-3′

Envelope gene-Reverse: 5′-GCGCAGTAAGGATGGCTAGT-3′

### Hematoxylin & eosin (HE) staining

Formalin-fixed paraffin-embedded mice lung tissue sections were deparaffinized by xylene and alcohol. The slides were then counterstained with hematoxylin for 15 min and eosin for 10 min. Then the tissue sections were dehydrated and mounted in the resinous medium.

### Multicolor immunofluorescence staining

Multicolor immunofluorescence analyses were performed using SARS-CoV-2 infected cells and lung tissues from patient with COVID-19. In brief, slides were dewaxed, followed by antigen retrieval in citrate buffer. Then the samples were permeabilized with 0.5% TritonX-100. After blocked with 5% goat serum, the slide was sequentially incubated with anti-spike (40150-R007, Sino Biological, China), anti-CD147 (Jiangsu Pacific Meinuoke Biopharmceutical Co. Ltd, China), anti-ACE2 (80031-R003, Sino Biological, China), anti-Rab5 (3547s, Cell Signaling Technology, USA), and anti-CD3 (ab5690, Abcam, UK) antibodies. TSA-Indirect Kit (PerkinElmer, USA) was used according to the manufacturer’s manual. Images were analyzed with Heilo software.

### Statistical analysis

Data from ELISA were analyzed using the parameter logistic curve. Significant differences were analyzed by unpaired *t* tests or Mann–Whitney *U* test with a two-tailed distribution. *p* < 0.05 was considered to be statistically significant. All experiments presented in this study yielded reproducible results for a minimum of three independent replicates. Statistical analyses were performed using the SPSS software (version 23.0) and GraphPad Prism software (version 8.0).

## Data Availability

The data sets in this study are available from the corresponding author upon reasonable request.

## References

[1] Jiang S, Xia S, Ying T, Lu L (2020). A novel coronavirus (2019-nCoV) causing pneumonia-associated respiratory syndrome. Cell. Mol. Immunol..

[2] Liu K (2020). Clinical characteristics of novel coronavirus cases in tertiary hospitals in Hubei Province. Chin. Med. J..

[3] Xu Z (2020). Pathological findings of COVID-19 associated with acute respiratory distress syndrome. Lancet. Respir. Med..

[4] Zhang, L. et al. The D614G mutation in the SARS-CoV-2 spike protein reduces S1 shedding and increases infectivity. Preprint at https://www.ncbi.nlm.nih.gov/pmc/articles/PMC7310631/ (2020).

[5] Hulswit RJ, de Haan CA, Bosch BJ (2016). Coronavirus spike protein and tropism changes. Adv. Virus Res.

[6] Lan J (2020). Structure of the SARS-CoV-2 spike receptor-binding domain bound to the ACE2 receptor. Nature.

[7] Hoffmann M (2020). SARS-CoV-2 cell entry depends on ACE2 and TMPRSS2 and is blocked by a clinically proven protease inhibitor. Cell.

[8] Qi F, Qian S, Zhang S, Zhang Z (2020). Single cell RNA sequencing of 13 human tissues identify cell types and receptors of human coronaviruses. Biochem. Biophys. Res. Commun..

[9] Daly JL (2020). Neuropilin-1 is a host factor for SARS-CoV-2 infection. Science.

[10] Cantuti-Castelvetri L (2020). Neuropilin-1 facilitates SARS-CoV-2 cell entry and infectivity. Science.

[11] Li Y (2009). HAb18G (CD147), a cancer-associated biomarker and its role in cancer detection. Histopathology.

[12] Lu M (2018). Basolateral CD147 induces hepatocyte polarity loss by E-cadherin ubiquitination and degradation in hepatocellular carcinoma progress. Hepatology.

[13] Pushkarsky T (2001). CD147 facilitates HIV-1 infection by interacting with virus-associated cyclophilin A. Proc. Natl Acad. Sci. USA.

[14] Zhang MY (2018). Disrupting CD147-RAP2 interaction abrogates erythrocyte invasion by *Plasmodium falciparum*. Blood.

[15] Zhao P (2011). HAb18G/CD147 promotes cell motility by regulating annexin II-activated RhoA and Rac1 signaling pathways in hepatocellular carcinoma cells. Hepatology.

[16] Bernard SC (2014). Pathogenic *Neisseria meningitidis* utilizes CD147 for vascular colonization. Nat. Med..

[17] Chen Z (2005). Function of HAb18G/CD147 in invasion of host cells by severe acute respiratory syndrome coronavirus. J. Infect. Dis..

[18] Gu J (2005). Multiple organ infection and the pathogenesis of SARS. J. Exp. Med..

[19] Tay MZ, Poh CM, Renia L, MacAry PA, Ng L (2020). The trinity of COVID-19: immunity, inflammation and intervention. Nat. Rev. Immunol..

[20] Yao H (2013). Important functional roles of basigin in thymocyte development and T cell activation. Int. J. Biol. Sci..

[21] Urra JM, Cabrera CM, Porras L, Rodenas I (2020). Selective CD8 cell reduction by SARS-CoV-2 is associated with a worse prognosis and systemic inflammation in COVID-19 patients. Clin. Immunol..

[22] Harrison SC (2015). Viral membrane fusion. Virology.

[23] Slonska, A., Cymerys, J. & Banbura, M. W. Mechanisms of endocytosis utilized by viruses during infection. Postepy Hig. Med. Dosw. (*Online*) 572–580 (2016). 10.5604/17322693.1203721 27333927

[24] Eyster CA (2009). Discovery of new cargo proteins that enter cells through clathrin-independent endocytosis. Traffic.

[25] Maldonado-Baez L, Cole NB, Kramer H, Donaldson JG (2013). Microtubule-dependent endosomal sorting of clathrin-independent cargo by Hook1. J. Cell. Biol..

[26] Saitoh S (2017). Rab5-regulated endocytosis plays a crucial role in apical extrusion of transformed cells. Proc. Natl Acad. Sci. USA.

[27] Zhai P (2020). The epidemiology, diagnosis and treatment of COVID-19. Int. J. Antimicrob. Agents.

[28] Li W (2003). Angiotensin-converting enzyme 2 is a functional receptor for the SARS coronavirus. Nature.

[29] Tipnis SR (2000). A human homolog of angiotensin-converting enzyme. Cloning and functional expression as a captopril-insensitive carboxypeptidase. J. Biol. Chem..

[30] Wong DW (2007). Loss of angiotensin-converting enzyme-2 (Ace2) accelerates diabetic kidney injury. Am. J. Pathol..

[31] Rentzsch B (2008). Transgenic angiotensin-converting enzyme 2 overexpression in vessels of SHRSP rats reduces blood pressure and improves endothelial function. Hypertension.

[32] Der Sarkissian S (2008). Cardiac overexpression of angiotensin converting enzyme 2 protects the heart from ischemia-induced pathophysiology. Hypertension.

[33] Kuba K, Imai Y, Ohto-Nakanishi T, Penninger JM (2010). Trilogy of ACE2: a peptidase in the renin-angiotensin system, a SARS receptor, and a partner for amino acid transporters. Pharm. Ther..

[34] Verdecchia P, Cavallini C, Spanevello A, Angeli F (2020). The pivotal link between ACE2 deficiency and SARS-CoV-2 infection. Eur. J. Intern. Med..

[35] Tan L (2020). Lymphopenia predicts disease severity of COVID-19: a descriptive and predictive study. Signal Transduct. Target Ther..

[36] Barth K, Blasche R, Kasper M (2006). Lack of evidence for caveolin-1 and CD147 interaction before and after bleomycin-induced lung injury. Histochem. Cell. Biol..

[37] Kuba K (2005). A crucial role of angiotensin converting enzyme 2 (ACE2) in SARS coronavirus-induced lung injury. Nat. Med..

[38] Wang K (2019). Identification of differentially expressed genes in non-small cell lung cancer. Aging.

[39] Nie J (2020). Establishment and validation of a pseudovirus neutralization assay for SARS-CoV-2. Emerg. Microbes Infect..

[40] Zhang L (2011). Morphology and structure of lipoproteins revealed by an optimized negative-staining protocol of electron microscopy. J. Lipid Res..

[41] Grigorieff N (2007). FREALIGN: high-resolution refinement of single particle structures. J. Struct. Biol..

[42] Frank J (1996). SPIDER and WEB: processing and visualization of images in 3D electron microscopy and related fields. J. Struct. Biol..

[43] Tang G (2007). EMAN2: an extensible image processing suite for electron microscopy. J. Struct. Biol..

[44] Ludtke SJ, Baldwin PR, Chiu W (1999). EMAN: semiautomated software for high-resolution single-particle reconstructions. J. Struct. Biol..

[45] Ohi, M., Li, Y., Cheng, Y. & Walz, T. Negative staining and image classification - powerful tools in modern electron microscopy. *Biol. Proced. Online* 23–34 (2004). 10.1251/bpo70 PMC389902 15103397

[46] Almabouada F (2013). Adiponectin receptors form homomers and heteromers exhibiting distinct ligand binding and intracellular signaling properties. J. Biol. Chem..

